# Structural equation modeling of the association between oral health literacy and dental caries in children

**DOI:** 10.1590/1807-3107bor-2025.vol39.021

**Published:** 2025-02-21

**Authors:** Larissa Chaves Morais de LIMA, Érick Tássio Barbosa NEVES, Matheus França PERAZZO, Veruska Medeiros Martins BERNARDINO, Samara Ellen da SILVA, Saul Martins de PAIVA, Fernanda de Morais FERREIRA, Ana Flávia GRANVILLE-GARCIA

**Affiliations:** (a) Universidade Estadual da Paraíba – UEPB, Department of Dentistry, Campina Grande, PB, Brazil.; (b) Universidade Estadual da Paraíba – UEPB, Department of Dentistry, Araruna, PB, Brazil.; (c) Universidade Federal de Goiás – UFG, School of Dentistry, Department of Social Dentistry, Goiânia, GO, Brazil.; (d) São Leopoldo Mandic – SL Mandic, Department of Odontopediatrics, Campinas, SP, Brazil.; (e) Universidade Federal de Minas Gerais – UFMG School of Dentistry, Department of Pediatric Dentistry and Orthodontics, Belo Horizonte, MG, Brazil.

**Keywords:** Health Literacy, Dental Caries, Pediatric Obesity, Parents, Oral Health, Latent Class Analysis

## Abstract

The aim of this study was to explore the association between oral health literacy (OHL) and dental caries in children, evaluating the direct and indirect effects of brushing frequency, obesity, and socioeconomic factors. A cross-sectional study was conducted with 739 schoolchildren aged eight to ten years and their parents/caregivers who answered a questionnaire addressing sociodemographic characteristics and oral hygiene habits as well as the OHL – Adult Questionnaire. Cavitated dental caries in the schoolchildren was evaluated using International Caries Detection and Assessment System criteria. Descriptive analysis was performed, followed by structural equation modeling into the theoretical model (95% CI). Goodness-of-fit indices were considered satisfactory (root mean square error of approximation < 0.06; comparative fit index > 0.90–0.95; standardized root mean square residual < 0.10 and Tucker-Lewis Index > 0.90–0.95). Mother’s age (standardized coefficient [SC]: -0.08; p < 0.01), caregiver’s schooling (SC: -0.22; p < 0.01), obesity (SC: 0.13; p < 0.01), and brushing frequency (SC: -0.09; p < 0.01) had a direct effect on dental caries, whereas OHL had an indirect influence on the outcome. Mother’s age, caregiver’s schooling, brushing frequency, and obesity directly affected the occurrence of cavitated carious lesions in children in the mixed dentition phase, whereas OHL had an indirect effect on this clinical outcome.

## Introduction

Dental caries is a chronic, multifactorial disease with a prevalence of 3.5 billion people throughout the world, and cavitated lesions can cause considerable functional, esthetic, and social problems in children.^1^ This oral condition results from the demineralization of the dental surface by bacteria due to organic acids in the diet^2^ and is also related to biological, physical, environmental and behavioral factors.^3^ Thus, associations between direct/indirect biopsychosocial determinants and dental caries should be considered in scientific analysis in order to reduce disparities in oral health.^4^

Unfavorable socioeconomic status is one of the factors that can affect the prevalence of cavitated dental caries, such as inadequate parental school level and low family income, which can limit access to dental services and exert a negative influence on oral health practices.^5^ Aspects such as the use of dental services, which may be influenced by the number of oral health teams close to children’s homes, also need to be considered, as easy access to dentists could improve the patient’s perception of oral health, satisfaction with dental care, and motivation for self-care.^6^

Oral health literacy (OHL) is a social and structural determinant that indicates the ability to process and understand information to improve and maintain oral health. It is also an aspect that has drawn the attention of researchers and health professionals and is considered to be a strong predictor of dental caries. Better OHL allows individuals to develop skills that facilitate the assimilation, understanding and adaptation of healthy practices, consequently reducing the occurrence of risk behaviors.^7^ Studies have shown that adequate OHL on the part of parents/caregivers translates to better oral health status in children, fewer harmful oral habits, better communication with health care providers and a better understanding of health care information.^8^

Previous studies have been conducted to assess the association between obesity and dental caries, but the results remain inconclusive.^9,10^ Studies show that genetic and environmental circumstances mediate the balance between intake of sucrose-rich foods and energy expenditure, which can lead to obesity.^11,12^ This health condition is highly prevalent in the population and is considered a serious public health problem throughout the world.^13^ Obesity and dental caries have common risk factors, and studies report the influence of social environment, emotional disorders, unhealthy eating habits, and economic factors on both conditions.^14,15^

The mixed dentition phase is a phase of important dental and skeletal transformations and in which children often have habits that are harmful for oral health,^16^ such as using a bottle or pacifier, high sugar consumption, and poor brushing.^1,10^ Furthermore, this age group is little explored in dental literature. Therefore, studying aspects directly and indirectly associated with dental caries in this period makes it possible to identify inadequate oral health habits and could help prevent functional and psychological harm.^3,6^

The hypothesis of the present study is that the OHL of parents/caregivers has an effect on dental caries in children and is mediated by oral hygiene habits. Thus, the aim of this study was to explore the association between OHL and dental caries in children, evaluating the direct and indirect effects of brushing frequency, obesity, and socioeconomic factors.

## Methods

### Ethical considerations

This research was approved by the Human Research Ethics Committee of Universidade Estadual da Paraíba (certificate number: 10514619.2.0000.5187) and is in accordance with the Declaration of Helsinki. Participating children signed a term of assent agreeing to participate and all parents/caregivers signed a statement of informed consent authorizing the participation of the children.

### Study design and sample

A population-based cross-sectional research was conducted in private and public schools in the city of Campina Grande and was reported following the recommendations of Strengthening the Reporting of Observational Studies in Epidemiology (STROBE statement) between February and November of 2019.^17^.The city has 30,467 students aged between eight and ten years enrolled in the school system and is divided into six administrative districts with a total of 131 schools (73 public and 58 private). The sample selection was probabilistic and performed by cluster in two stages (schools and students) stratified by administration district. Firstly, 23 schools were randomly selected using Microsoft Excel program (Microsoft Office 365, Microsoft, Redmond, WA, USA) to ensure the representativeness of the sample. In the second stage, schoolchildren were selected using a simple random sampling method proportional to the number of students enrolled in each district until the number of children necessary to reach the desired sample size was obtained.

The sample size for analytical comparison studies was calculated with two independent proportions using the G* Power program, version 3.1 (Franz Faul, Universitat Kiel, Germany), adopting a 95% significance level and 80% power. The proportion estimates from the pilot study indicated caries rates of 74.1% and 26.9% in children whose parents had low and high levels of OHL, respectively. The minimum sample was determined to be 396 children. A design effect of 1.6 was applied to increase the variability of the sample, leading to 634 students, and 20% was then added to compensate for possible dropouts. Thus, the desired sample size was 760 students. However, there was a sample loss of 3.79% and the final sample of this work was 739 children.

### Eligibility criteria

Schoolchildren aged 8 to 10 years in the mixed dentition phase and enrolled in public and private schools, in addition to their parents/caregivers, were included in the study. Children with syndromes or neurological disorders, those who took anticonvulsant or antidepressant medications, and those who wore fixed orthodontic appliances were excluded. The information was previously reported by teachers, parents, or caregivers before clinical examination, so these children were not considered for the research.

### Calibration and pilot study

Cavitated dental caries were diagnosed using the International Caries Detection and Assessment System (ICDAS) by four calibrated research dentists. The calibration involved a theoretical step and a practical step under the orientation of an experienced examiner who served as the gold standard. For the practical step, 40 children from a public school selected for convenience were examined and re-examined after 7 days for intra-examiner agreement. The Kappa statistic revealed good inter-examiner and intra-examiner agreement (Kappa > 0.80). All researchers were also trained for questionnaire administration.

The pilot study was conducted with 30 children (15 from a private school and 15 from a public school). The schools and children participating in the calibration process and pilot study were selected for convenience and did not participate in the main study. This step revealed that there was no need to alter the methods.

### Data collection

Information about the socioeconomic questionnaire was explained to the teachers and students, such as that the questions should be answered with a pen and that no questions should be left blank. Parents that could not read should ask a family member or the student’s teacher for help. The questionnaires were delivered to the children so that the parents/caregivers could answer them at home. The questionnaire on sociodemographic characteristics addressed the age of the parents/caregivers and the child, child’s sex and skin color, mother’s schooling, monthly family income and number of residents in the home. The age of the parents/caregivers was also one of the variables of interest. Child related questions like daily toothbrushing frequency, use of a fixed device, and the presence of syndromes or neurological disorders were also addressed. The number of oral health teams in the child’s geographic district was obtained from the municipal Secretary of Health.

The Oral Health Literacy – Adult Questionnaire (OHL-AQ) was answered by parents/caregivers individually at home without help from others. The OHL-AQ was validated for the Brazilian context and is composed of 22 items divided in four sections: reading comprehension, numeracy, listening, and decision making. Reading comprehension consists of three incomplete sentences on oral health knowledge. The participant must read the questions and choose one of four possible answers for each question. The interviewer must not help the participants to read, answer, or understand the meaning of the items, but can explain how to fill out the questionnaire and examine whether there are any missing items, giving the participant the opportunity to answer the item or select the “I don’t know” option. Correct answers are scored one point and incorrect answers are scored zero points. The total score is the sum of the item scores and ranges from 0 to 17, with higher scores denoting a higher level of OHL. The scores were used in the structural modeling and, for descriptive analysis, OHL was categorized as inadequate (0–9 points), marginal (10–11 points), or adequate (12–17 points). ^22^

The children performed supervised brushing and were then examined individually at the school. The examiners used personal protective equipment, a head lamp with a light-emitting diode (Petzl Zoom; Petzl America, Clearfield, USA), a sterilized mouth mirror (PRISMA, São Paulo, Brazil), sterilized Williams probe (OMS-621; Trindade, Campo Mourão, Brazil), and gauze to dry the teeth, following the methods indicated by the World Health Organization.^22^

Cavitated tooth decay was assessed using the ICDAS. This index has a scoring system based on the extent of the carious lesion with codes ranging from 0 (sound) to 6 (extensive cavity exposed at base and on walls). Due to the epidemiological nature of the study, scores “1” and “2” were grouped (code 2), given the impossibility of drying with an air jet. Teeth that presented scores ≥ 3 were classified as cavitated caries. A severe disease was considered for scores five and six, due caries lesion reaching the dentin^20^.

1. = healthy, immediately after drying with gauze; no caries, stain, hypoplasia, wear, erosion, and other non-carious phenomena.
2. = Immediately after drying, first visible change in the enamel or changes in color limited to pits and fissures.
3. = Observation without drying, distinguishable visual change, white or colored, to an extent that goes beyond the pits and fissures.
4. = Localized enamel rupture, no visible dentin, discontinuity on the enamel surface.
5. = Underlying dark shadow from the dentin, with or without localized enamel breakdown.
6. = Cavity with exposed dentin at the base of the cavity.
7. = Extensive, visible cavity in dentin, at the base and walls (more than half of the surface).

Anthropometric data were analyzed with the aid of the WHO AnthroPlus software (2007) for children and adolescents 5 to 19 years of age, although our sample consisted of schoolchildren aged 8 to 10 years. The data used by the program are child’s weight, height, birthdate, and sex and date of the clinical examination. Separate tables and graphs are used for each indicator. Z-scores and a percentile classification are used for the interpretation of the results. In the present study, quantitative scores were used, with a higher percentile indicating a greater degree of obesity.^22^

### Statistical analysis

Descriptive analysis was used for sample characterization, and categorical variables were expressed as absolute frequencies and percentages, whereas continuous variables were expressed as mean and standard deviation (SD) using SPSS for Windows (version 22.0; IBM Corp., Armonk, USA). In addition, structural equation modeling was used for the determination of direct and indirect associations between variables in the conceptual model, adopting a 95% confidence interval (CI) in the Mplus program, version 6.11. Recent studies available in the literature^6,19,20^ were used as basis for the theoretical model. The models that reliably tests hypothesis were evaluated using standardized coefficients (factor loadings) and goodness-of-fit indices: root mean square error of approximation (RMSEA < 0.06); comparative fit index (CFI > 0.90–0.95); Tucker-Lewis Index (TLI > 0.90–0.95); and standardized root mean square residual (SRMR < 0.10). Pathway analysis was conducted using the MPlus statistical package, version 8.8.

## Results

The final sample consisted of 739 children aged eight to ten years, corresponding to a 94% response rate. Girls accounted for 50.3% of the sample. Most mothers had more than eight years of schooling (57.5%) and were younger than 35 years (77.7%). More than two-thirds of the parents/caregivers had inadequate or marginal OHL (71.7%). The mean number of teeth with cavitated lesions was 2.2 (SD = 2.8). The mean number of oral health teams in the child’s district was 5.9 ± 1.6, and 51.8% of the children resided in a district with more than five oral health teams. Table 1 displays the absolute and relative frequencies of the variables used instructural model.

**Table 1:** Absolute and relative frequencies of the variables used in structural model.

Variables	n (%)
Sex	
Male	367 (49.7)
Female	372 (50.3)
Child’s age (years)	
8	369 (36.4)
9	240 (32.5)
10	230 (31.1)
Guardian’s schooling	
≤ 8 years of study	310 (42.2)
> 8 years of study	425 (57.5)
Mother’s age (years)	
≤ 35	384 (52.7)
> 35	345 (47.3)
Monthly family income	
≤ R$ 1100 (minimum monthly wage)	327 (44.2)
> R$ 1100	247 (33.4)
Nutritional status	
Median (min/max)	18.2 (10.7–35.4)
Obesity	163 (22.1)
Ideal range	576 (77.9)
Daily brushing frequency	
Mean (SD)	1.8 (±0.8)
Up to 1x per day	270 (36.9)
2 to 3 x per day	574 (61.0)
More than 3x per day	150 (20.4)
Oral health literacy of parents/guardians	
Mean (SD)	10.2 (3.3)
Inadequate	255 (34.5)
Marginal	275 (37.2)
Adequate	209 (28.3)
Quantity of oral health teams in child’s district	
Mean (SD)	5 (1.6)
< 5	356 (48.2)
5 to 8	383 (51.8)
Number of tooth surfaces with cavitated lesions – Mean (SD)	2.2 (±2.8)
Presence of dental caries	
Yes	390 (52.8)
No	349 (47.2)

Table 2 displays the structural equation model and goodness-of-fit criteria. The model demonstrated a good fit: x^2^/df = 1.61, CFI = 0.93; RMSEA = 0.04 (95% CI: 0.039-0.049); TLI = 0.92; and SRMR = 0.09.

**Table 2:** Estimated standardized effects for the structural equation model.

Variables	Standardized coefficients	
Pathways	Coefficient	p-value
Oral health literacy in		
Monthly family income	0.141	< 0.01
Guardian’s schooling	0.380	< 0.01
Mother’s age	0.015	0.71
Quantity of oral health teams	0.011	0.80
Dental caries in		
OHL	0.015	0.73
Sex	0.013	0.72
Obesity	0.131	< 0.01
Toothbrushing frequency	-0.098	< 0.01
Quantity of oral health teams	0.014	0.68
Guardian’s schooling	-0.228	< 0.01
Mother’s age	-0.088	< 0.01
Toothbrushing frequency in OHL		
Sex	0.106	<0.01
Quantity of oral health teams	0.051	0.25
Mother’s age	0.130	< 0.01
Obesity in	0.002	0.97
Sex		
Guardian’s schooling	-0.003	0.23
Mother’s age	0.01	0.81
Quantity of oral health teams in		
Monthly family income	0.128	< 0.01
Goodness-of-fit indices	Index	95% CI
CFI	0.938	-
TLI	0.92	
RMSEA	0.044	0.039-0–049
SRMR	0.090	-

OHL: oral health literacy; CFI: Comparative Fit Index; TLI: Tucker-Lewis Index.

Figure illustrates the theoretical reference of the study. Mother’s age (standardized coefficient [SC]: -0.08; p < 0.01), caregiver ’s schooling (SC: -0.22; p < 0.01), obesity (SC: 0.13; p < 0.01), and brushing frequency (SC: -0.09; p < 0.01) had a direct effect on prevalence of dental caries, whereas OHL, family income, and number of oral health teams in the child’s geographic district had an indirect influence on the outcome. The interpretation of the standardized coefficients was as follows: in the predictor changes by x standard deviation units when the result increases by one standard deviation controlling for group.

**Figure:**
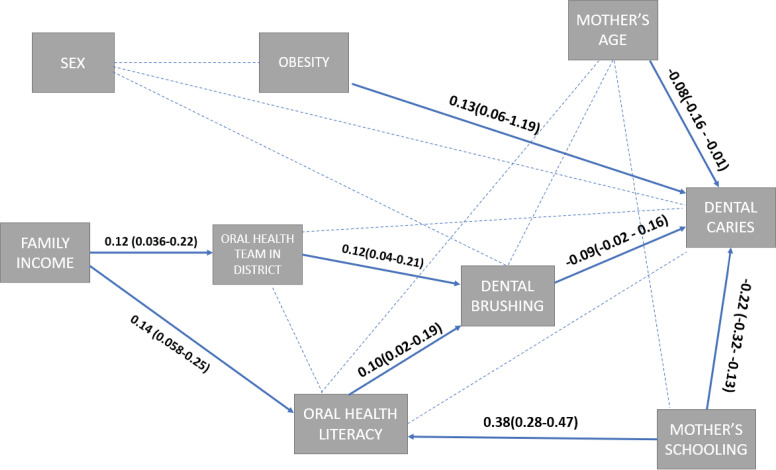
Conceptual model of the study including direct and indirect associations with respective standardized coefficients and 95% confidence intervals.

## Discussion

The present work evaluated direct and indirect factors associated with cavitated carious lesions in children in the mixed dentition stage. The conceptual hypothesis was confirmed. Parental OHL had an indirect association with the child’s dental caries, and this relationship was mediated by brushing frequency. Moreover, the child’s nutritional status, mother’s age, and caregiver’s schooling were directly associated with the clinical outcome. These findings are relevant and emphasize the importance of OHL as a social indicator of dental caries, which could help reduce oral health inequalities among children.^3^

In this study, the number of cavitated dental caries was greater in children whose mothers were younger and had a lower level of schooling. This finding is in agreement with data reported in previous studies, as these parents have less information on oral health, which can exert an influence on oral hygiene habits, consumption of cariogenic foods, dental care, and, consequently, on the prevalence of dental caries in their children.^21,22^ Previous studies have shown that a lower socioeconomic status limits the use of health care services and reduces access to information on prevention and the adoption of prevention practices.^23^

The mean OHL score of the parents/caregivers was 10.3 ± 3.3 and only 28.3% of the respondents had an adequate level of this construct. OHL had an indirect effect on the prevalence of dental caries mediated by toothbrushing frequency. Low OHL limits the understanding of patient-dentist communication, which can be an obstacle to maintaining good oral hygiene practices, resulting in dental caries.^7,24,25^ A previous study employed structural equation modeling to investigate the relationship between OHL and dental caries among 12-year-old adolescents and found a direct association.^3^ However, toothbrushing frequency was not evaluated and the tool used for OHL assessment was based only on word recognition on the part of the children rather than the understanding and interpretation of parents/caregivers.

Obesity was directly associated with cavitated carious lesions. This effect may be explained by the negative influence of obesity on living habits, such as oral hygiene and a high-sugar diet, which directly affect oral health.^24,26^ The association between biological indicators of oral diseases and nutritional status has also been investigated. Lower rates of stimulated saliva secretion,^27^ higher levels of inflammatory markers in the gingival crevicular fluid,^28^ and different oral microbial profiles, which are potential indicators of dental caries, were found in individuals with obesity.^29^ However, these relationships have been little investigated in children and the results of systematic reviews remain inconclusive.^10,15,30^

Results of this nature are important, as the mixed dentition phase is characterized by facial growth and changes in dental arches.^4^ Moreover, cavitated carious lesions in the primary dentition tend to persist in the permanent dentition and have a negative impact on oral health-related quality of life. Thus, the early diagnosis of factors associated with this clinical condition is fundamental for the prevention of functional and psychosocial effects.^31^

Direct and indirect associations with dental caries were investigated using validated instruments, a representative population-based sample, and statistical analysis involving structural equation modeling, which is a reliable and robust approach to this topic in children. The STROBE statement was followed in this research, minimizing the possibility of bias.^17^ The results are important and can help to strengthen measures to promote health literacy and knowledge on healthy eating in the primary education setting. Such findings could also be used by other studies to clarify direct and indirect factors associated with dental caries in this age group.

## Conclusion

In conclusion, mother’s age, caregiver’s schooling, toothbrushing frequency, and obesity directly affected the occurrence of cavitated carious lesions in children in the mixed dentition phase, whereas OHL of parents/caregivers had an indirect effect on this clinical outcome.

This study evaluated dental caries and the associated factors in schoolchildren, helping pediatric dentists in the early diagnosis and prevention of this oral disease. This work emphasizes the importance of evaluating direct and indirect factors associated with cavitated dental caries to carry out multidisciplinary educational programs.
